# Investigating the Feasibility of Processing Activated Carbon/UHMWPE Polymer Composite Using Laser Powder Bed Fusion

**DOI:** 10.3390/polym14163320

**Published:** 2022-08-15

**Authors:** Yas Khalil, Neil Hopkinson, Adam J. Kowalski, John Patrick A. Fairclough

**Affiliations:** 1 Department of Mechanical Engineering, University of Sheffield, Sheffield S3 7HQ, UK; 2 Stratasys Ltd., 5-6 William Lee Buildings, Science Park, Nottingham NG7 2RQ, UK; 3 Unilever plc, R&D Port Sunlight Laboratory, Wirral CH63 3JW, UK

**Keywords:** additive manufacturing, 3D printing, laser sintering, UHMWPE, activated carbon, polymer composite, composite characterisation, composite processing

## Abstract

Activated Carbon (AC) is widely available at a relatively low cost, has a high porosity and is commonly used as a filter material for a range of applications. However, it is a brittle and friable material. Ultra-High Molecular Weight Polyethylene (UHMWPE) polymer is a tough engineering plastic that has been used as a binder. The traditional method used in manufacturing AC/UHMWPE filters involves compressing AC/UHMWPE composite powder during heating in a mould. This process compresses the particles together and the materials undergo sintering. This process results in a low pore interconnectivity, which has a considerable impact on the filter’s efficiency. Selective Laser Sintering is a laser powder bed fusion additive manufacturing technique for polymers. This has a number of advantages compared to the conventional technique and produces a porous structure with improved filtration efficiency. We propose that this is due to the greater pore interconnectivity. In this work, AC/UHMWPE powdered composites were prepared with different AC and UHMWPE ratios. The structure and properties of the AC/UHMWPE composite were investigated and characterised to assess their suitability for selective laser sintering. Particle size and morphology analysis were conducted, as well as density measurements, powder flow, thermal analysis, and crystallinity measurements. The results reveal that the addition of AC improves the UHMWPE flow. The thermal analysis results show that the intrinsic thermal properties of UHMWPE powder are not significantly affected by the introduction of activated carbon. However, thermal gravimetric analysis revealed that the onset of mass loss is considerably shifted (20 °C) to higher temperatures for the AC/UHMWPE composites, which is favourable for laser sintering. Additionally, the change in the composition ratio of untreated composite does not have a significant effect on the degree of crystallinity. Laser-sintered AC/UHMWPE parts were successfully manufactured using a commercial laser-sintering machine.

## 1. Introduction

Activated carbon (AC) is commonly used as a filter bed material in air handling systems, respirator masks and chemical protection suits. The activation process increases the porosity and, therefore, the surface area, and changes the chemical nature of the carbon surface. However, AC materials show brittle failure at low loads. Thus, most filters either incorporate the AC with a binder or encase the AC in some form [1].

AC is produced from a wide range of carbon-containing organic materials, including coconut shells, wood, coal, fruit stones, sawdust, lignite, and peat. AC is an effective and reliable filter material. AC removes a range of impurities and has a large adsorptive capacity, including organic and chlorinated molecules. It can also be physically and chemically tailored to suit a particular application. AC is used in a wide range of applications including water and gas purification, air filters in gas masks and respirators, medicine, air pollution control, energy storage, fuel cells, batteries and many other applications [2,3,4,5,6].

Ultra-High Molecular Weight Polyethylene (UHMWPE) has many commercial applications, such as in food and beverages, foundry, chemical processing, mining, lumber, textile, paper, transportation and medicinal applications [1]. However, UHMWPE is difficult to process with conventional methods due to its high melt viscosity [7,8]. Therefore, powder processing techniques have been used as an alternative way to manufacture UHMWPE parts, including powder compression moulding and ram extrusion [9,10]. Although, the limited processing options restrict UHMWPE’s applications [8], the UHMWPE processing can be enhanced by the addition of high, thermally conductive fillers to the UHMWPE matrix [1], such as carbon fibre, carbon nanofibre, carbon nanotubes, graphite and carbon black [1,11,12]. Thus, AC/UHMWPE composites may provide complementary synergies in filter applications.

Selective laser sintering (SLS) is a well-established laser powder bed fusion additive manufacturing technique, which is used to produce end-use parts directly from computer-aided design data. The SLS process uses a powder-based material that is deposited layer by layer into a heated bed. The heated bed temperature is set below the melting point of the polymer. The parts are created layer by layer using additional heat generated from a high-power IR laser beam. This fuses adjacent particles (and, to some degree, layers) together. At present, there is a limited palette of materials for laser sintering. This is partly due to the complex thermal and consolidation phenomena that occur during the laser sintering process [13].

Many factors may influence the successful processing of a polymer in SLS, such as its powder properties, melting behaviour, thermal stability and crystallisation and solidification [14]. Powder properties, such as particle size, shape and powder flow, can affect the powder processing and the quality of the parts produced [15]. Khalil et al. [16,17,18] reported difficulties when processing UHMWPE using SLS. The irregular shapes of these non-conducting particles lead to highly agglomerated particles, poor packing (and, thus, low powder density), and poor powder flow. These factors mean that UHMWPE powder is unfavourable for laser sintering.

Differential Scanning Calorimetry (DSC) allows for the determination of the melting and crystallisation behaviour of a polymer. This is a useful tool for the evaluation of the polymers’ suitability for the sintering process. The phase transformations during melting and crystallisation are the key parameters. For materials that show a relatively sharp melting endotherm, the powder can be heated to a temperature just under the melting point throughout the sintering process cycle [19]. Another thermal characteristic required for successful sintering is to have a wide processing window [20], which is defined as the difference between the temperatures at the onset of melting on heating and the onset of re-crystallisation on cooling.

The Thermogravimetric Analysis (TGA) method is a useful tool for the compositional analysis of blends, thermal stability, moisture and volatile content [21]. One of the material requirements for laser sintering is the ability to withstand a high thermal load during the preheating stage and the laser application [18]. Therefore, analysing the thermal stability of the material is recommended to evaluate degradation and identify the various thermal transformations that occur during laser sintering.

The physical and mechanical properties of polymers are affected by the degree of crystallinity [22,23,24,25]. On the other hand, the addition of carbonaceous materials to polymers may influence the crystallinity and change these properties [26]. Therefore, it is imperative to determine the degree of crystallinity and examine whether any interaction occurs between the blend materials that alter the crystallinity. The degree of crystallinity in polymers can be determined using various methods, such as DSC and X-ray diffraction (XRD).

The aim of this study is to investigate material requirements and determine whether AC/UHMWPE composite powder has potential for selective laser sintering. In this work, three different AC and UHMWPE ratios were used to prepare the AC/UHMWPE powdered composites (70% AC to 30% UHMWPE, 80% AC to 20% UHMWPE and 85% AC to 15% UHMWPE (weight/weight)). These ratios were prepared similarly to the conventional method used in manufacturing AC/UHMWPE filters. The prepared composite was characterised by light scattering and scanning electron microscopy (SEM), for particle size and morphology analysis; helium gas pycnometry for density measurement; DSC and TGA for thermal analysis, and XRD for crystallinity measurement. Additionally, in order to confirm the feasibility of processing AC/UHMWPE powders, an attempt was made to laser sinter the AC/UHMWPE composite at different laser power levels using a commercial laser-sintering machine. The density and mechanical properties were evaluated and compared. Since this study is investigating the feasibility of processing an AC/UHMWPE composite using laser sintering, process optimisation was not carried out, but is recommended for future work.

## 2. Materials and Characterisation Methods

### 2.1. Materials and Preparation of AC/UHMWPE Composite Powder

The powders used in this study were UHMWPE and Activated Carbon. UHMWPE GUR 2122 (Celanese, Sulzbach, Germany) is a thermoplastic polyolefin material with extremely long chains and a molecular mass of around 4.5 × 10^6^ g/mol. UHMWPE of the GUR 2122 grade has a nodular morphology with a low powder bulk density of 0.20–0.25 g/cm^3^ (GUR^®^ 2122 PE-UHMW datasheet, Celanese, Germany). Powdered Activated Carbon produced from coconut shell with a mesh size of 60 × 200 (250–75 μm) was supplied by Eurocarb Products Ltd. (UK) and manufactured by high-temperature steam activation. AC product specification is listed in Table 1, as provided by the supplier (YAO 60 × 200 AW datasheet, Eurocarb, UK).

The UHMWPE and AC powders were sieved separately to remove large, agglomerated particles. UHMWPE powder was then dry-mixed with AC using a tumbler mixer (mixing station P1, EOS GmbH, Germany) for approximately 60 min. The mixing ratios used were 70% AC to 30% UHMWPE, 80% AC to 20% UHMWPE and 85% AC to 15% UHMWPE (weight/weight), as shown in Table 2.

### 2.2. Particle Size and Morphology

The particle size of UHMWPE and AC powders was determined by laser diffraction using a Mastersizer 3000 (Malvern Instruments, Malvern, UK). A dry powder-dispersion method was used to analyse the samples using air as media. A three-bar feed pressure with ten measurements per run was used to determine the distribution of the particle size.

Microstructure images of the AC/UHMWPE powder were captured using a scanning electron microscope (Philips XL-20, Philips, Eindhoven, Holland) at an accelerating voltage of 13 kV and with different magnifications. The sample was prepared by placing double-sided adhesive tape on the sample holder, which was then dipped in the powder. If there was any extra powder on the sample holder, then the holder was shaken to leave a small number of particles. A gold sputter coater was used before examining the sample.

### 2.3. Powder Density and Flow

The true density of AC, bulk and tapped densities of AC/UHMWPE powder were measured, and the powder flow was investigated.

For composite powders, densities were measured according to the rule of mixtures (Equation (1)). This assumes that a given composite property is the volume-weighed average of the two phases’ properties [27]. For density, this gives:

(1) $$\rho_{c}=f\times \rho_{\mathrm{UHMWPE}}+(1-f)\times \rho_{\mathrm{AC}}$$

where $\rho_{c}$, $\rho_{\mathrm{UHMWPE}}$ and $\rho_{\mathrm{AC}}$ are the densities of the composite, UHMWPE and AC powders, respectively, while *f* and (*1 − f*) are volume fractions of UHMWPE and AC powders, respectively.

#### 2.3.1. True, Bulk and Tapped Densities

A helium gas pycnometer (AccuPyc II 1340, Micromeritics, Norcross, GA, USA) was used to measure the true density of AC and UHMWPE powders.

The powder bulk density was determined by the mass of the powder per unit volume. The volume was measured by pouring the powder into a predefined volume container and allowing it to settle under the effect of gravity [18]. Then, the mass of the powder in the container was weighed.

The tapped density was determined by tapping the container holding the powder using a tapping mechanism. After 500 taps, the container’s contents were weighed, and the tapped density was calculated. This method is described in Khalil et al., (2019) [18].

#### 2.3.2. Powder Flow

The powder flow was assessed using Hausner’s method, which is based on the ratio between the tapped density and bulk density. This ratio is known as ‘Hausner Ratio (HR)’, which classifies the flow of powders as follows [18,28]:

- If the HR value is less than 1.25, then the powder should have a high flow.
- If the HR value is between 1.25 and 1.4, then a reduced powder flow is expected.
- When the HR value is greater than 1.4, then the powder is considered to be cohesive.

Powders with an HR under 1.25 (i.e., high flow) are considered to be suitable powders for laser sintering, with adequate density [28]. The layer density of the powder in the powder bed can be affected by the powder flow and packing. Some interaction between the particles occurs when the powder flows easily, resulting in a high density. The porosity in the laser-sintered parts decreases with a high layer density, and this improves the part quality and accuracy [29].

The measurement of the bulk and tapped densities were repeated three times on each sample, the Hausner ratio was calculated, and the average values were taken.

### 2.4. Differential Scanning Calorimetry (DSC)

The thermal properties of the composite powders were measured at a heating rate of 10 °C/min by DSC (DSC 8500, PerkinElmer, Waltham, MA, USA) from 25 to 220 °C under a nitrogen atmosphere. DSC curves in AC/UHMWPE composites were obtained under two thermal cycles, and three samples were tested for each set of parameters. The weight of the samples was 7 mg on average.

### 2.5. Thermogravimetric Analysis (TGA)

This analysis aimed to determine the amount of UHMWPE and activated carbon used in the composites powders and to examine the influence of the composition on the thermal stability of the composites.

TGA was performed using TGA analyzer (Pyris 1 TGA, PerkinElmer, Waltham, MA, USA) to investigate the thermal stability of AC/UHMWPE composite. A material sample of approximately ten milligrams was heated from 25 °C to 600 °C at a heating rate of 10 °C/min under nitrogen atmosphere.

The extrapolated onset temperature, which refers to the temperature at which the weight loss begins, is the intersecting point of two lines drawn tangent to the two linear regions of the TGA curve. The onset temperature was used for consistency. This temperature is also recommended by both ASTM E1131 and ISO 11358 standards. The delta Y, the change in mass, was used to measure the “as run” sample and determine the mass fractions of each phase.

### 2.6. X-ray Diffraction Analysis (XRD)

A Bruker D8 Advance X-ray diffractometer (Bruker AXS GmbH, Karlsruhe, Germany) with a Cu Kα anode with a wavelength (λ) of 0.15418 nm was used for XRD analysis. The system operated at 40 kV and 40 mA. The AC/UHMWPE powder samples were mounted on the stage and scanned from 10° to 70° using a step size of 0.05° and time per step of 10 s.

### 2.7. Degree of Crystallinity

The estimated degree of crystallinity of AC/UHMWPE composite powders was determined using DSC and XRD methods. The effect of mass fraction of activated carbon on the overall crystallinity was investigated.

#### 2.7.1. Differential Scanning Calorimetry

The estimated overall degree of crystallinity of AC/UHMWPE powders was determined using the DSC method. The degree of crystallinity (*X*_C_) was calculated using the following equation [23,25,30]:

(2) $$X_{c}(\%)=(\frac{\Delta H}{\Delta H_{100}\times \left(1-W_{f}\right)})\times 100$$

where ∆*H* is the melting enthalpy of the AC/UHMWPE composite powder, ∆*H*_100_ is the melting enthalpy of polyethylene containing 100% of crystallinity, which is 293 J/g [9], and *W_ƒ_* is the weight fraction of activated carbon. Three DSC measurements were averaged, and the overall crystallinity of the samples was estimated.

#### 2.7.2. X-ray Diffraction

The diffraction peaks at around 15°–30° of the AC/UHMWPE composites were a mixture of the diffraction peaks in neat UHMWPE and AC. The crystallinity was calculated using the area of crystalline peaks in the region of 2θ between 15 and 30° and the area of the diffuse background of the amorphous in this region, which was estimated using a Gaussian function. The crystallinity was then calculated using the following equation:

(3) $$X_{c}(\%)=(\frac{Ac}{A_{C}+A_{A}})\times 100$$

where *A_C_* and *A_A_* are the areas of crystalline phase and amorphous phase, respectively.

## 3. Results and Discussion

### 3.1. Powder Particle Size and Morphology

**Figure 1:**
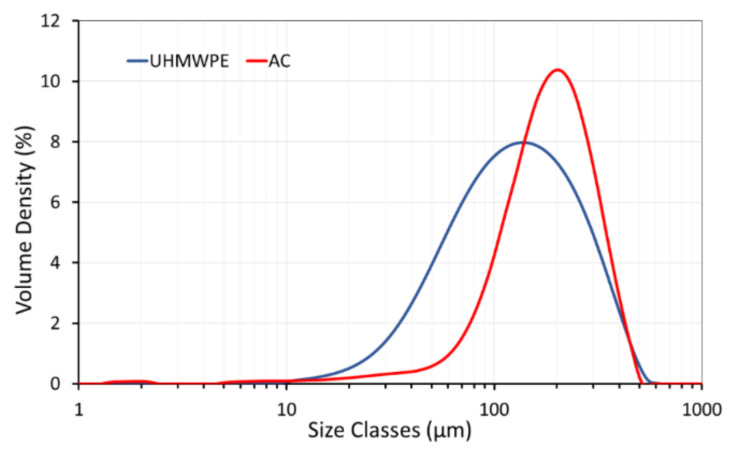
Particle size distribution of AC and UHMWPE powders.

The powders used for SLS require a certain particle size distribution that enables better powder flowability. Typical commercial-grade SLS polymer materials have a particle size between 45 μm and 90 μm, which is favourable for laser sintering [31]. A high number of fine particles may induce agglomeration and reduces powder flowability, which is unfavourable for SLS processing [32]. Figure 1 shows the particle size distribution of the AC and UHMWPE powders. The average particle size of AC and UHMWPE powders was 196 μm (d_50_) and 125 μm (d_50_), respectively. AC and UHMWPE powders exhibit narrow particle size distributions, with AC being relatively broader than UHMWPE. The small particle size can fill the spaces between the irregular-shaped particles in the AC/UHMWPE composite, leading to reduced interactions or agglomerations and consequently enhancing the flowability [33].

**Figure 2:**
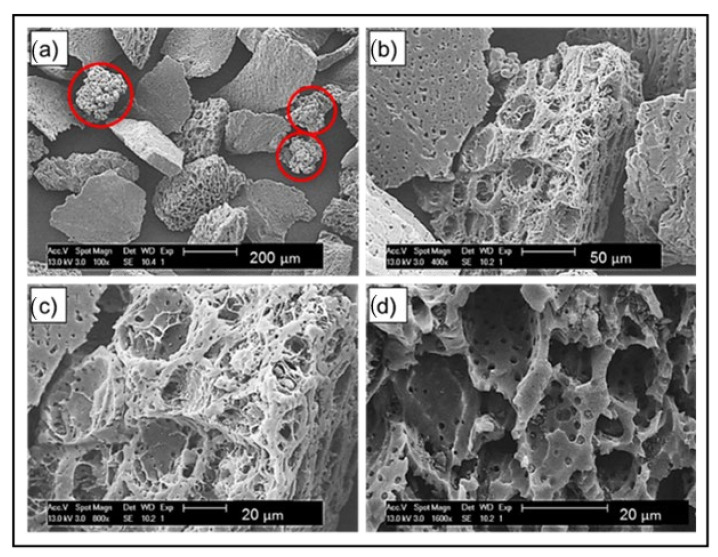
SEM images of unsintered AC/UHMWPE powder. (**a**) UHMWPE particles are highlighted in red surrounded by irregular AC particles (100×); (**b**) AC particles showing a range of surface structures (400×); (**c**) open structure of some ACs (800×); (**d**) open structure AC with large and small pores (1600×).

Figure 2 shows the textural and morphological properties of AC/UHMWPE composite powder. The highly porous AC particles have an irregular shape with a wide variety of pores. Well-developed large pores with honeycomb shapes could be observed on the surface of the particles. The coarse particles of the AC powder may enhance the flow compared to fine powders [33].

### 3.2. Composite Powder Density and Flow

The true density values of UHMWPE and AC powders, measured by the helium gas pycnometry method, were 0.954 ± 0.0016 g/cm^3^ and 2.1718 ± 0.0677 g/cm^3^, respectively. The results of the measurements of the densities (true, bulk and tapped) and Hausner ratios are listed in Table 3.

**Figure 3:**
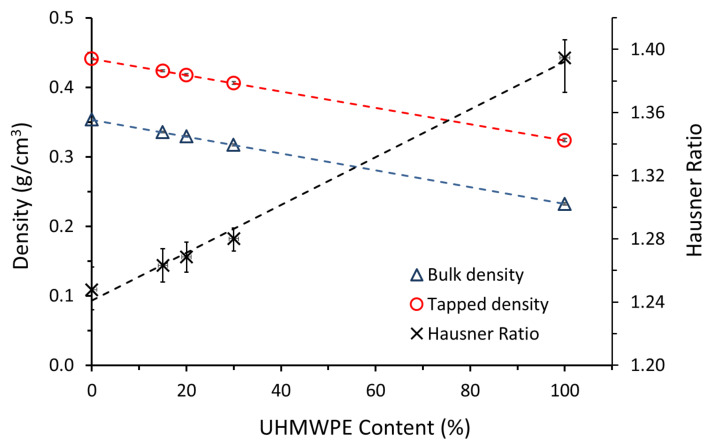
Bulk and tapped densities and Hausner ratios of AC/UHMWPE composites.

Figure 3 shows that the bulk and tapped densities of the composite powders increase with increasing amounts of activated carbon, as expected.

Based on the criteria of distinguishing between cohesive and non-cohesive powder, (Section 2.3.2), the Hausner ratios of the powders for PE100, PE30, PE20, PE15 and AC100 were determined to be 1.39, 1.28, 1.27, 1.26 and 1.25, respectively (Table 3 and Figure 3). This suggests that the AC powder has high flow and the addition of AC improved the flow of UHMWPE (original HR = 1.39). This improvement was observed during the spreading and distribution of the powder in the laser-sintering machine. It is evident that the HR does not strictly follow the “rule of mixtures”, in that it is not a simple linear relationship with regard to concentration. This is probably due to the increased AC-AC particle contact.

The powder flow can be influenced by a large number of variables, including the particle size, shape and surface roughness [34]. Coarse powders (large particle size) flow more easily than fine powders [33]. This influence was evident in this case, as the particle size of AC was larger than the size of UHMWPE particles (Figure 1). Abdullah and Geldart [35] suggested that the flow of the powder increases with the increase in particle size, but above a certain optimum, the flow does not show further improvement.

### 3.3. Differential Scanning Calorimetry

**Figure 4:**
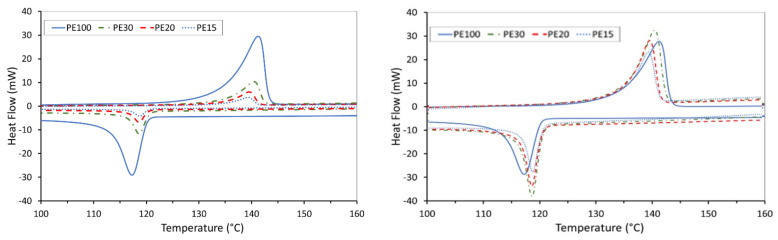
DSC curves of the neat UHMWPE and AC/UHMWPE composites (1st Run). (**Left**) raw data; (**Right**) data normalised by the mass of UHMWPE within the sample mixture.

**Figure 5:**
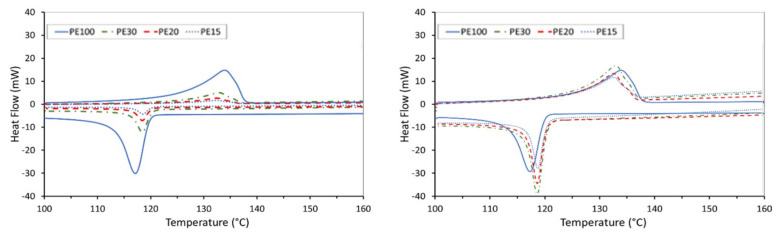
DSC curves of the neat UHMWPE and AC/UHMWPE composites (2nd Run). (**Left**) raw data, (**Right**) data normalised by the mass of UHMWPE within the sample mixture.

The thermal properties of the neat UHMWPE and the AC/UHMWPE composite powders are summarised in Table 4 (melt and crystallisation peaks) and the DSC curves of the first and second thermal cycling are presented in Figure 4 and Figure 5, respectively.

**Table 4:** Thermal properties of neat UHMWPE and composite powders.

	1st Thermal Cycle			2nd Thermal Cycle		
Sample ID	Melting Point Peak (°C)	Crystallisation Point Peak (°C)	ΔH (melt) * (J/g)	Melting Point Peak (°C)	Crystallisation Point Peak (°C)	ΔH (Melt) * (J/g)
PE100	142 ± 0.8	118 ± 0.2	173 ± 8.0	134 ± 0.8	117 ± 0.3	128 ± 0.2
PE30	143 ± 0.3	121 ± 0.1	175 ± 7.0	136 ± 0.1	121 ± 0.1	131 ± 5.8
PE20	143 ± 0.1	121 ± 0.1	168 ± 17.1	136 ± 0.3	121 ± 0.1	120 ± 15.0
PE15	142 ± 0.1	121 ± 0.2	149 ± 6.0	136 ± 0.2	121 ± 0.2	105 ± 5.0

* Data was normalised for the mass of UHMWPE.

The results show that the intrinsic thermal properties of UHMWPE powder are not significantly affected by the introduction of activated carbon. A slight change was observed, but this is most likely due to the powered nature of the materials. This results in poor thermal contact between the powder and the sample pan, particularly for the AC that does not melt. This result indicates that there was no interaction between the UHMWPE and AC materials at the targeted temperature. The small DSC sample size (7 mg), coupled with the variability in the mixing, may lead to discrepancies in the DSC peak area within the mass normalised data.

### 3.4. Thermogravimetric Analysis

**Figure 6:**
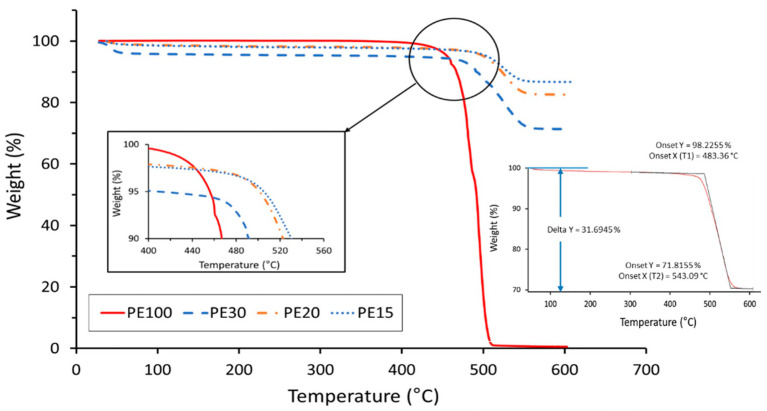
TGA results for AC/UHMWPE composite powders.

TGA curves in neat UHMWPE and AC/UHMWPE composites are presented in Figure 6, and the corresponding thermal characteristic data are listed in Table 5.

**Figure 7:**
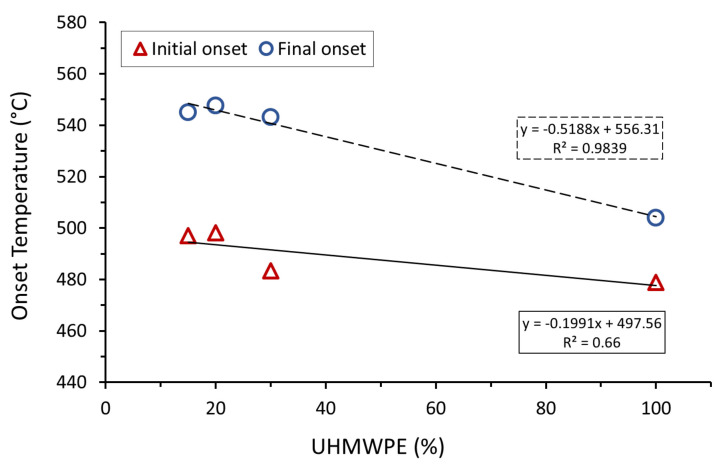
Effect of AC addition on the thermal stability of UHMWPE.

The initial mass lost below 100 °C is due to moisture. The thermal degradation of the neat UHMWPE starts at around 478°C. The results show that the onset of mass loss considerably shifts to higher temperatures for the AC/UHMWPE composites (Figure 7). For composites containing 15% and 20% of UHMWPE, this shift was around 20 °C. Similar behaviour was observed with Carbon Nanofibre-Reinforced Ultrahigh Molecular Weight Polyethylene [36]. The sample mass of the composites remained constant at temperatures above 550 °C, effectively allowing for a verification of the actual UHMWPE content.

The TGA determines the mass lost. As activated carbons can adsorb the volatile degraded UHMWPE components during the degradation process, there are two possible explanations for the observed results. Either the thermal stability of the AC/UHMWPE increased with the increase in the AC content, which may be due to the AC absorbing the volatile gases from the UHMWPE and retarding degradation, or the mass lost as volatile gas by the polymer was taken up by the activated carbon, resulting in minimal mass change. TGA is not able to differentiate between these two scenarios. A similar TGA study by Watts et al. shows that the oxidation of polyethylene is retarded by carbon nanotubes; this increases the oxidative stability by 18 °C [37]. However, these were intimately mixed in the melt. Here, they are separated as individual grains of powder.

### 3.5. X-ray Diffraction Analysis

**Figure 8:**
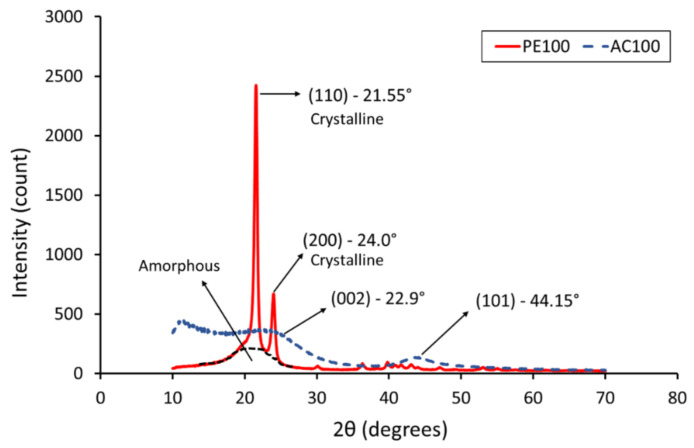
XRD profile of neat UHMWPE and activated carbon powders.

The XRD spectrum for UHMWPE and AC powders is shown in Figure 8. The XRD pattern of AC shows broad peaks, and the absence of sharp peaks suggests a predominantly amorphous structure, which is a typical characteristic of AC [38,39]. Two broad peaks were observed at 2θ = 22.9° and 2θ = 44.15°, which correspond to the reflections of (002) and (101) planes, respectively [15].

**Figure 9:**
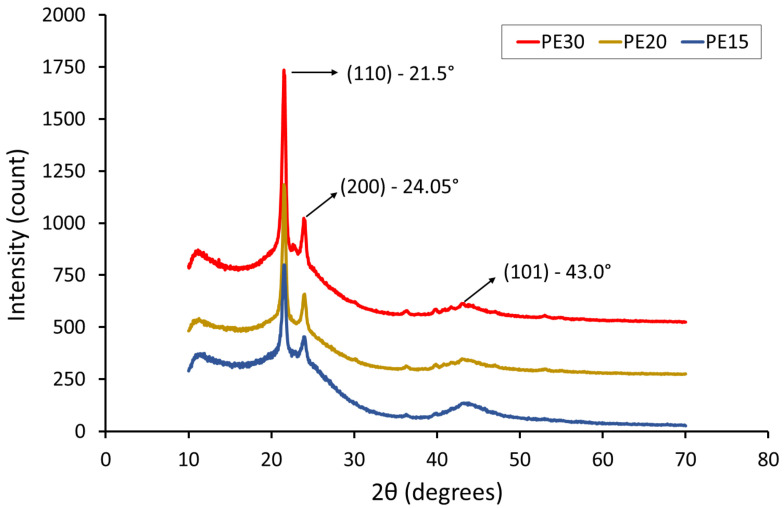
XRD profile of AC/UHMWPE composite powders. The traces have been vertically shifted for clarity.

The XRD patterns of AC/UHMWPE composites PE30, PE20 and PE15 are shown in Figure 9. Two sharp peaks can be observed at 2θ = 21.5° and 24.05°, which were assigned to the (110) and (200) reflections, respectively [40]. The broad range of 2θ angles covering between 16 and 26° occurred as a result of the amorphous phase (AC and UHMWPE) [41]. The sharp peaks in (110) and (200) crystal planes were 21.5°, 21.5°, 21.55° and 24.05°, 24°, 24° for PE30, PE20 and PE15, respectively, The XRD profiles of the AC/UHMWPE composites show a new weak and wide diffraction peak near 2θ = 43.0°, which is assigned to the (101) diffraction planes of graphitic carbon coming from activated carbon and confirming its amorphous state [8].

The results show that there is no noticeable difference in the XRD patterns of the AC/UHMWPE composites (PE30, PE20 and PE15) and no obvious shift in diffraction peak positions can be observed. This result suggests that the ratio of the unprocessed AC/UHMWPE composite does not affect the microstructure of the composite materials.

### 3.6. Degree of Crystallinity

The estimated degree of crystallinity of AC/UHMWPE powder was determined using DSC and XRD methods. The effect of mass fraction of activated carbon on the overall crystallinity was investigated.

**Figure 10:**
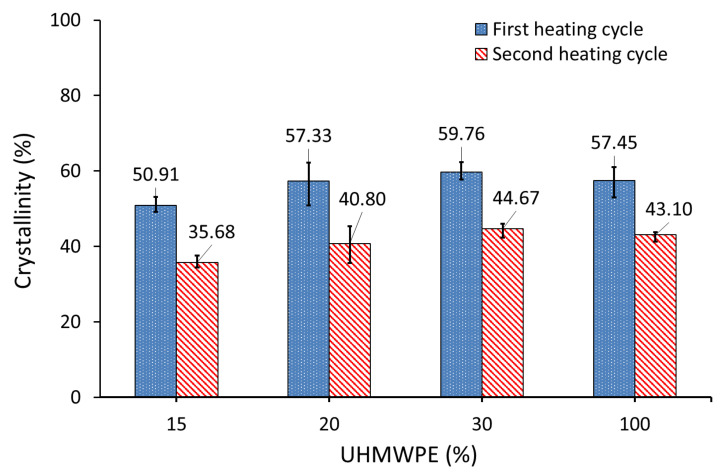
Degree of crystallinity of AC/UHMWPE composites measured by DSC.

DSC measurements were performed to determine the crystallinity of the AC/UHMWPE composite powders (i.e., PE30, PE20 and PE15). All the data were normalised for the mass of UHMWPE. Figure 10 shows that the enthalpy of melting and, therefore, the calculated crystallinity of the first heating cycle, slightly increased with the decrease in the mass fraction of activated carbon. 

This result was unexpected, since the tests were performed on untreated powders (virgin powders). The composite powders were mixed mechanically, and no interaction should occur between the composite materials under the specified test temperature; therefore, the crystallinity of untreated AC/UHMWPE composites should remain unchanged with the change in the composite content ratios.

One of the reasons for the observed disagreement could be related to the inconsistency caused by the manual mixing method and sampling error when preparing DSC samples. However, these changes in crystallinity could be also influenced by the higher thermal conductivity of the activated carbon and poor contact between the AC powder and DSC pan. The key to obtaining good data in this scenario is minimising the effect of non-uniform temperature distribution, and this can be achieved by coating the aluminium pan with a thin coating of silicon oil before placing the powder in the pan.

The DSC measurements were repeated, and the composite powder samples were directly placed in the DSC pan, after coating it with a thin layer of a silicone oil to improve the thermal contact between the pan and the test sample. The pan in the reference side was also coated with silicone oil.

**Figure 11:**
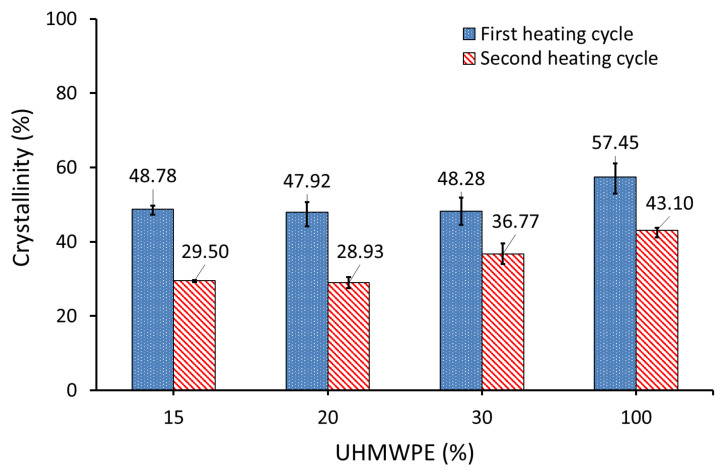
Degree of crystallinity of AC/UHMWPE composites measured by DSC. DSC pan was coated with silicone oil to improve thermal contact.

Figure 11 shows that the degree of crystallinity of AC/UHMWPE composites did not change in the first heating cycle with the change in the mass fraction of activated carbon. This result confirmed our initial prediction regarding the crystallinity behaviour of untreated AC/UHMWPE composites. Silicon oil was chosen as it does not mix with UHMWPE and is not expected to change crystallisation. However, the degree of crystallinity in the second heating cycle of AC/UHMWPE composites decreased with the increasing mass fraction of activated carbon.

Table 6 shows the degree of crystallinities of AC/UHMWPE composite powders, calculated from the first heating (X_C1_) and second heating (X_C2_) cycles of the DSC, and the crystallinity obtained by XRD measurements (X_C3_).

The degree of crystallinity of the neat UHMWPE (PE100) decreased from 57.45% to 48.28% (using DSC) and from 67.80% to 61.35% (using XRD) for AC/UHMWPE composite with 70% AC and 30% UHMWPE (PE30). The three AC/UHMWPE composite samples (i.e., PE30, PE20 and PE15) showed statistically insignificant changes in crystallinity, confirming that the change in the composition ratio of an untreated composite does not have a significant effect on the degree of crystallinity. This slight change is most likely due to sampling or measurement errors.

From the degree of crystallinity values presented in Table 6, it was observed that the crystallinities calculated from XRD and DSC are not in good agreement. The average values of the XRD results were higher than that of the DSC (first heating cycle) by 10.35%, 13.07%, 9.23% and 7.32% for the PE100, PE30, PE20 and PE15 powders, respectively. The degree of crystallinity values calculated using DSC and XRD may differ from one another for a number of reasons, including the sample size and the random selection of the sample from the bulk powder used in DSC, as well as the differences in the way the background and amorphous scattering are determined in XRD, which introduces some subjectivity to the calculation. Generally, the crystallinity measured by the XRD method shows higher values compared to the DSC method. This is a widely known issue in polymer systems, as these methods measure two different properties [42].

## 4. Laser-Sintering Trials of AC/UHMWPE Composite

A commercial laser-sintering system EOS Formiga P100 was used to manufacture test samples from AC/UHMWPE powder. Before sintering the test samples, a range of processing conditions were attempted so suitable parameters could be obtained to process the parts. Based on the experience gained from sintering UHMWPE, a laser power of 8 watts and laser scan speed at 2500 mm/s, with a range of powder bed temperatures starting from 135, 125, 120, 117.5, 115 and 110 °C, were initially used to find suitable bed temperatures, achieve a good powder flow and produce multilayer parts. As the removal chamber temperature does not have a significant effect on the process or the part quality, this was set to the same temperature as the bed for all trials. A layer thickness of 0.1 mm, laser count of 2 (double scan) and hatch spacing of 0.15 mm were fixed for all trials. The laser-sintering parameters selected for the initial trials are listed in Table 7. The composite virgin powder used for the initial trial was 70% AC and 30% UHMWPE (wt/wt).

**Figure 12:**
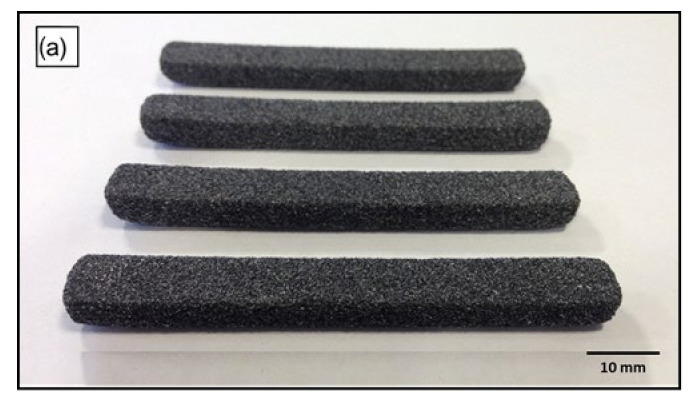

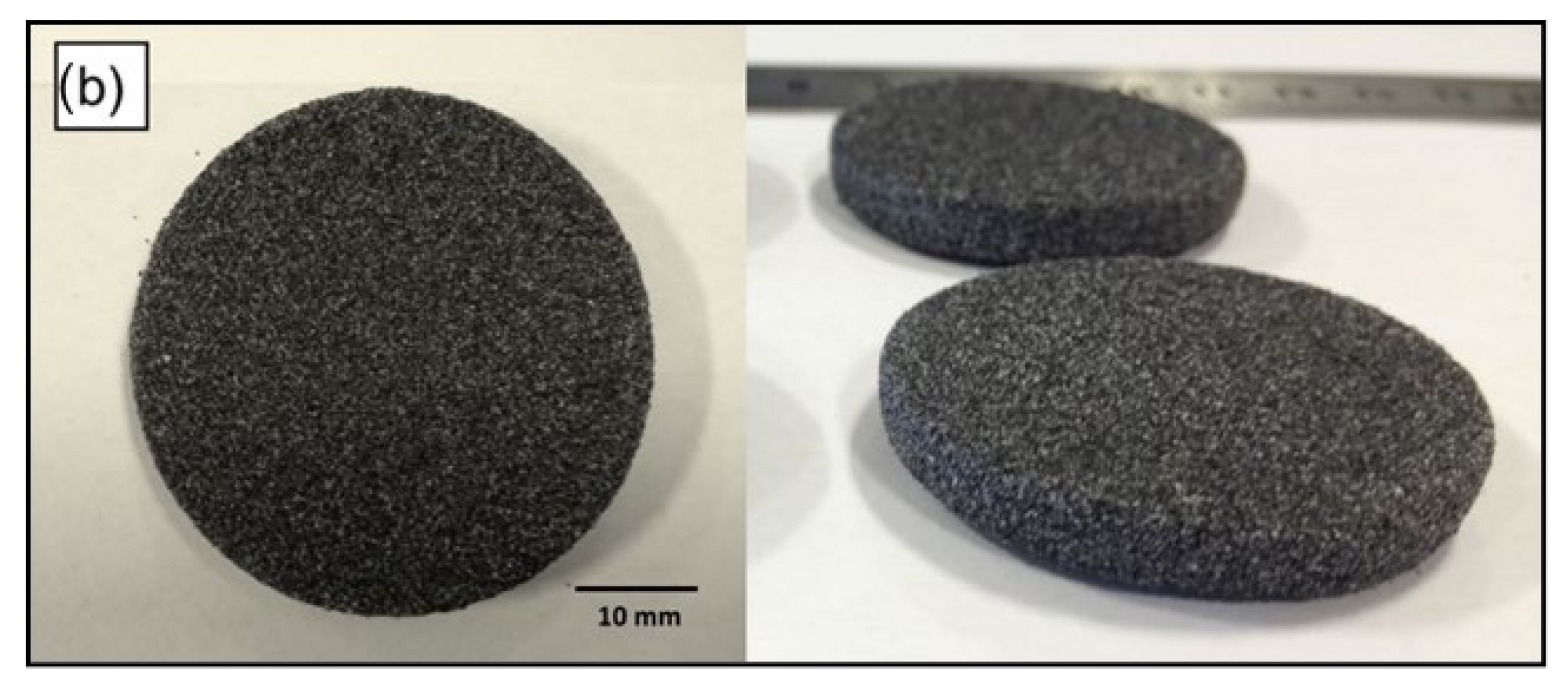
AC/UHMWPE laser-sintered parts produced during the trials at a bed temperature of 117.5 and a laser power of 8 watts. (**a**) Rectangular-shaped samples, all the samples are the same size (80 × 10 × 4 mm). (**b**) Disc-shaped samples (20 mm radius and 4 mm thickness).

The laser sintering process and sintered parts of the initial trials were visually assessed, and also assessed by manual handling of the parts. Bed temperatures between 110 °C and 120 °C were found to be suitable in terms of powder spreading, reducing the effect of curling, producing multilayer parts, ease of powder removal and cleaning. However, observations during laser sintering showed that the composite powder flows much better at bed temperatures of 117.5 °C and 120 °C. Additionally, powder removal was easier at these temperatures. Figure 12 shows that AC/UHMWPE parts were produced with good definition and structural integrity.

After identifying a suitable bed temperature, new samples were produced with laser powers of 6, 8, 10 and 12 watts to establish the range of laser powers that produce well-defined multilayer parts with good mechanical properties. The laser-sintering parameters selected for this test are listed in Table 8. A fixed bed temperature of 117.5 °C was selected for all samples.

**Figure 13:**
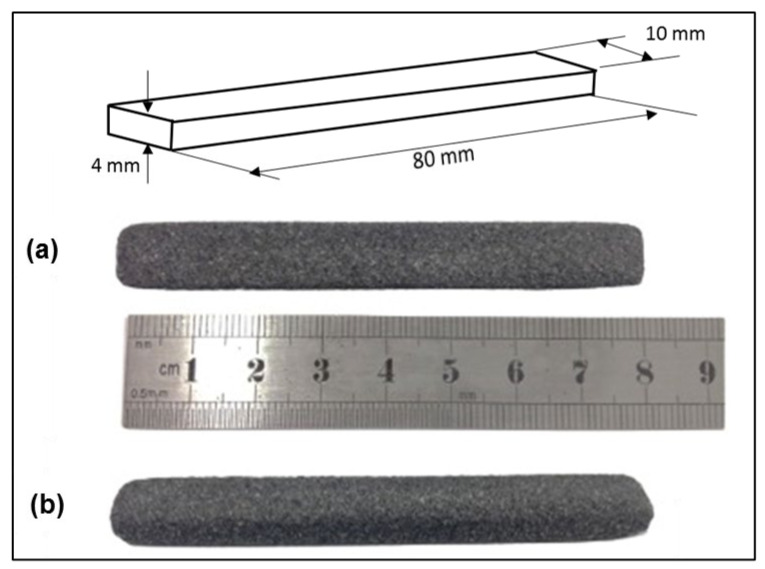
Laser-sintered AC/UHMWPE flexural test samples produced at laser power of 12 watts: (**a**) top view, (**b**) side view.

Laser-sintered parts with a length of 80 mm, width of 10 mm and thickness of 4 mm (according to ISO 178), were built in x–y orientation with the long axis parallel to the x-axis, width parallel to the y-axis and thickness parallel to z-axis (i.e., build direction). Laser-sintered AC/UHMWPE parts were successfully manufactured using different laser powers with well-defined structures and good mechanical properties (Figure 13).

### 4.1. Relative Density

**Figure 14:**
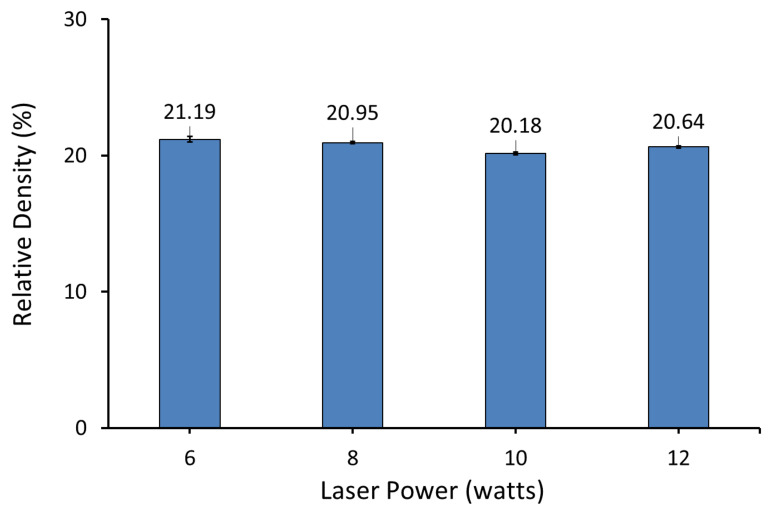
Relative density of AC/UHMWPE (70%/30%) produced at various laser powers and bed temperature of 117.5 °C.

Relative density is the ratio of the bulk density of the parts to the density of the material composing the parts (i.e., theoretical density or true density of the materials that make up the powder). The results presented in Figure 14 show the relative density of AC/UHMWPE (70%/30% wt/wt) samples produced at laser powers of 6, 8, 10 and 12 watts and a bed temperature of 117.5 °C.

Results showed statistically insignificant changes in the relative density with the change in laser power in the range between 6 and 12 watts. This result suggests that the density of the laser-sintered AC/UHMWPE parts is mainly governed by the AC and the changes in laser power did not significantly alter the overall density of the parts.

### 4.2. Mechanical Properties

**Figure 15:**
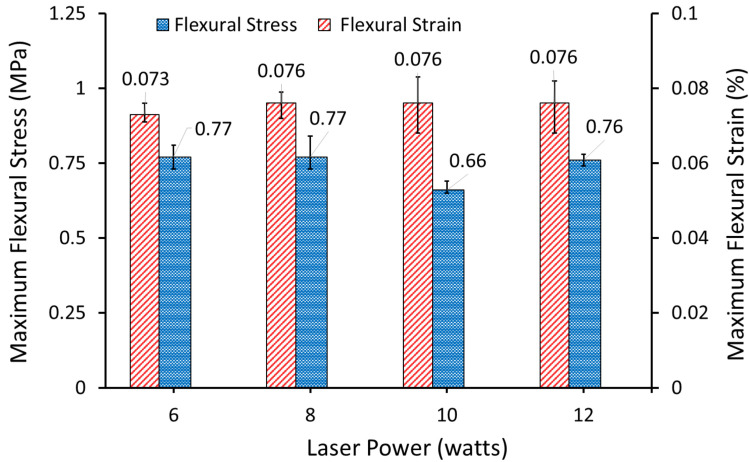
Maximum Flexural Stress and Strain of AC/UHMWPE (70/30%) produced at various laser powers and bed temperature of 117.5 °C.

A Texture Analyser (TA500, Lloyd Instruments, West Sussex, UK) fitted with a 50 N load cell was used to determine the flexural properties of the sintered AC/UHMWPE samples. The test machine was run with a constant crosshead speed of 0.4 mm/min and span length of 40 mm for all samples. Figure 15 shows the maximum flexural stress and strain of AC/UHMWPE (70%/30% wt/wt) samples produced at laser powers of 6, 8, 10 and 12 watts and a bed temperature of 117.5 °C.

The results suggest that the flexural stress remains unchanged within a laser power range between 6 and 12 watts. However, a slight drop in flexural stress can be observed at laser power of 10 watts and which is likely due to statistical variation.

Similarly, the average flexural strain remains unchanged within a laser power range between 6 and 12 watts. A flexural strain with an overall average of 0.075 ± 0.002% was achieved; this shows that the flexural properties are dominated by the relatively brittle AC and not the tough UHMWPE. This result showed statistically insignificant changes in the flexural strain with the change in laser power, at the specified range. Using different laser powers did not significantly alter the overall flexural strain of the sintered parts.

## 5. Conclusions

The results show that the AC has a high powder flow and the addition of AC has improved the flow of UHMWPE. The flow and packing are influenced by the shape and the friction between particles. Since the friction increases with the increase in the specific surface area of the powder, then using coarser particles would induce better flow than fine powders due to the high inter-particle friction between the particles of the fine powders [38].

The DSC results show that the intrinsic thermal properties of UHMWPE powder are not significantly affected by the introduction of activated carbon, indicating that no interaction occurred between the UHMWPE and AC materials at the target temperature.

The TGA results revealed that the onset of mass loss shifted to considerably higher temperatures for the AC/UHMWPE composites. For the composites containing 15 and 20% of UHMWPE, this shift was around 20 °C. This suggests that the thermal stability of the AC/UHMWPE increased with the increase in the content of the activated carbon. Activated carbons can preferentially adsorb the volatile degraded UHMWPE components during the degradation process. This could delay the mass loss and/or retard the thermal degradation, resulting in a higher perceived degradation temperature for the composites compared to that of the neat UHMWPE. The result clearly shows that the temperature window from DSC and TGA is sufficiently wide and favourable for laser sintering.

As indicated by the XRD data, there is no noticeable difference in the XRD patterns of the AC/UHMWPE composites, and no obvious shift in diffraction peak positions was observed. This suggests that the ratio of the unprocessed AC/UHMWPE composite does not affect the microstructure of the composite materials.

Similarly, the degree of crystallinity of the three AC/UHMWPE composite samples (i.e., PE30, PE20 and PE15) showed statistically insignificant changes in the trend confirming that the change in the composition ratio of untreated composite does not have a significant effect on the degree of crystallinity. However, a slight change was observed, which is most likely due to sampling or measurement error.

This work has demonstrated that, for the first time, 3D-printed AC/UHMWPE parts can be produced using selective laser sintering. Trials to laser sinter non-standard SLS materials (i.e., off-the-shelf powders) of AC/UHMWPE composite were carried out. Parts with well-defined structure were successfully manufactured at various laser powers using a commercial laser-sintering machine: EOS Formiga P100. For future work, it is highly recommended to investigate the influence of process parameters and composite composition on the material characteristics and mechanical properties of the laser-sintered AC/UHMWPE parts.

## Figures and Tables

**Table 1:** Powdered Activated Carbon specification.

Particle Size (µm)	Bulk Density (g/cm^3^)	Surface Area (m^2^/g)	Ash Content (%)	pH	Moisture Content (%)
75–250	0.440–0.490	1250	1 (max)	6–8	5 (max)

**Table 2:** Powder sample labels.

Sample Labels	PE100	PE30	PE20	PE15	AC100
**UHMWPE (wt%)**	100	30	20	15	0
**AC (wt%)**	0	70	80	85	100

**Table 3:** Densities and Hausner ratios of composite materials.

Composite Ratios	True Density (g/cm^3^)	Bulk Density (g/cm^3^)	Tapped Density (g/cm^3^)	Hausner Ratio (HR)
PE100	0.954	0.2321	0.3236	1.39 ± 0.02
PE30	1.8065	0.3170	0.4057	1.28 ± 0.01
PE20	1.9282	0.3291	0.4175	1.27 ± 0.01
PE15	1.9891	0.3352	0.4233	1.26 ± 0.01
AC100	2.1718	0.3534	0.4409	1.25 ± 0.01

**Table 5:** TGA analysis thermal data of neat UHMWPE and AC/UHMWPE composites.

	Onset Temperature (°C)		UHMWPE Mass (%)		
Sample ID	T_1_ *	T_2_ *	Theory	Actual (ΔY)	Actual (Onset)
PE100	478.84	503.92	100	99.48	98.75
PE30	483.36	543.09	30	31.70	26.39
PE20	498.18	547.61	20	17.35	15.01
PE15	497.03	545.03	15	13.33	10.77

* T1 and T2 are the initial and final decomposing temperatures, respectively. The variation in the actual mass loss, (ΔY), compared with the expected mass loss, is due to the small sample size in TGA and the variation in sample mixing.

**Table 6:** Degree of crystallinity of neat UHMWPE and AC/UHMWPE composite powders.

	Crystallinity (%)—DSC		Crystallinity (%)—XRD
Sample ID	X_C1_	X_C2_	X_C3_
PE100	57.45 ± 3.70	43.10 ± 1.24	67.80 ± 8.50
PE30	48.28 ± 3.70	36.77 ± 3.86	61.35 ± 3.65
PE20	47.92 ± 3.40	28.93 ± 1.51	57.15 ± 2.95
PE15	48.78 ± 1.34	29.50 ± 0.31	56.10 ± 0.20

**Table 7:** Laser-sintering parameters of the initial trials.

Parameter	Unit	Initial Range
Laser Power	watt	8
Bed Temperature	°C	110, 115, 117.5, 120, 125, 135
Removal Chamber Temperature	°C	110, 115, 117, 120, 125, 135
Laser Scan Speed	mm/s	2500
Hatch Spacing	mm	0.15
Laser Count	-	2 (double scan)
Layer Thickness	mm	0.1

**Table 8:** Laser-sintering parameters used to identify suitable laser power range for the initial trials.

Parameter	Unit	Range
Laser Power	watt	6, 8, 10, 12
Bed Temperature	°C	117.5
Removal Chamber Temperature	°C	117
Laser Scan Speed	mm/s	2500
Hatch Spacing	mm	0.15
Laser Count	-	2 (double scan)
Layer Thickness	mm	0.1

## Data Availability

The data presented in this study are available on request from the corresponding author.
